# FEDECOM: Enabling cross-border energy exchange by federating energy communities

**DOI:** 10.12688/openreseurope.19146.1

**Published:** 2024-12-19

**Authors:** Zia Lennard

**Affiliations:** 1 R2M Solution SAS, Roquefort-les-Pins, France

**Keywords:** Cross-border energy exchange, Energy sector coupling, Energy communities, Renewable energy, Cloud-based energy management, FEDECOM platform, Peer-to-peer energy trading

## Abstract

The FEDECOM project (Federated-system approach for flexible and interoperable energy communities) is a European Union-funded project aimed at fostering the integration and flexibility of local energy systems by demonstrating the potential of cross-border energy exchange by energy sector coupling through a federation of energy communities. The project seeks to provide economic benefits, improve grid stability and reliability, and contribute to the decarbonisation of the energy system. FEDECOM combines direct and indirect electrification strategies to leverage synergies and offers a cloud-based platform for managing local energy systems, including power, gas, thermal energy, industry, and mobility sectors. This paper discusses FEDECOM’s approach to energy management at multiple pilot sites, seeking to optimize resource use, enhance grid stability, and promote sustainable energy practices. It presents an overview of the FEDECOM project's goals, structure, pilot sites, and technical innovations, including peer-to-peer trading, semantic data modeling, and a novel platform architecture.

## 1. Introduction

Energy systems across Europe are transitioning towards increased sustainability, flexibility, and local autonomy, supported by recent policy directives such as the European Green Deal. FEDECOM, a collaborative EU-funded project, addresses this transition by focusing on federated energy communities and sectoral coupling to improve grid stability, economic viability, and decarbonization. Specifically, the project is developing innovative solutions for a sustainable energy system, addressing regulatory barriers, promoting digitalization through a cloud-based platform and advanced data analytics, and creating new market opportunities for energy communities, service providers, and technology developers, contributing to economic growth and job creation.

## 2. Objectives and scope

FEDECOM is a consortium of international organizations working to develop a cloud-based platform that uses Information and Communication Technologies (ICT) to promote energy efficiency and sectoral coupling. The platform will use Distributed Energy Resources (DER) and storage systems in pilot sites with varying equipment and climatic conditions. The project aims to enable energy sector coupling, integrate local energy systems, optimize resource management, and develop partner-backed plans for large-scale replication. FEDECOM's scientific and technical objectives include optimizing operations, promoting efficient energy consumption and generation, promoting peer-to-peer energy trading, and promoting a more sustainable and resilient energy infrastructure. The project's activities range from creating tools for peer-to-peer trading to optimizing access to the energy market and renewable energy (RE) use.

## 3. Methods

### 3.1 Platform architecture and key functionalities

The FEDECOM platform consists of 14 modular components, each designed to address specific energy community needs. Key functionalities include forecasting ( section 3.1.1), optimal energy management ( section 3.1.2), peer-to-peer (P2P) energy trading ( section 3.1.3), a semantic data model ( section 3.1.4), and a Multi-Criteria Decision Analysis (MCDA) recommender ( section 3.1.5). Forecasting involves prosumer generation, while optimal energy management uses real-time dispatching considering supply and demand sides. Demand response optimizes local energy systems and unlocks 30% of demand side flexibility. The platform also facilitates energy and flexibility trading within and between communities, ensuring transparency and transparency in contractual frameworks.

***3.1.1 Forecasting module*.** The FEDECOM platform's Forecasting Module is A real-time generation forecasting tool for prosumer resources that leverages historical and real-time data to predict production and consumption levels across communities, facilitating efficient energy dispatch and demand response. It is designed to provide accurate predictions of both RE generation and energy demand. This module will utilize a variety of techniques, including physical models, data-driven forecasting methods, and machine learning algorithms, to generate forecasts over different time horizons, ranging from one hour to seven days ahead. RE generation forecasts will be based on factors such as weather data, while energy demand forecasts will incorporate household, building, and community-level profiles. The Forecasting Module will also estimate the share of flexible load, providing valuable input for the optimization of energy systems. The accuracy of these forecasts will be assessed using metrics such as Root Mean Square Error (RMSE). The development of forecasting algorithms for RE generation and thermal demand is already underway, with initial algorithms being tested and validated using historical datasets. These forecasts are essential for informing the operation of other FEDECOM modules, such as the Model Predictive Control (MPC), enabling efficient energy management and maximizing the utilization of RE sources (RES).

***3.1.2 Optimal Energy Management System (EMS)*.** The FEDECOM platform's Optimal Energy Management System is an integrated solution designed to manage energy communities effectively. It leverages a suite of predictive, modeling, and optimization capabilities to facilitate cost-optimized management of energy assets connected to the communities. The system monitors various data points from energy assets, including building thermal behavior and energy loads, storage system status, RE generation, and potential energy flexibility of each asset. This information is then processed by different modules within the platform, such as the Energy Dispatch Model, Grid Analytics & Diagnostics, and MPC Service. These modules work together to analyze the data and propose optimized scenarios for energy asset usage within the community. The system also enables peer-to-peer energy and energy flexibility trading within and between communities using a blockchain-based local marketplace, facilitated by the Open Marketplace module. The system aims to ensure efficient, stable, and reliable grid operation while promoting energy savings and economic benefits for the community.

***3.1.3 Peer-to-Peer energy trading*.** The FEDECOM platform facilitates P2P energy trading within and between energy communities using a blockchain-based local marketplace, aiming to optimize local energy use and grid resilience. This marketplace, built on the Energy Web Chain and Substrate-based blockchain tools, ensures secure data flow, data integrity, and transparent transactions through immutable distributed ledger mechanisms. FEDECOM's P2P trading concept allows community members to buy and sell excess RE generation, prioritizing self-consumption and minimizing reliance on the traditional grid. This localized trading can reduce grid congestion and network charges, particularly when energy exchange occurs within the same distribution network. The platform supports different P2P trading models: third-party to third-party, energy communities to third parties, and energy communities to energy communities. The latter model fosters a network of energy communities exchanging energy based on complementary production and consumption patterns, maximizing local RE utilization and supporting grid stability. While the RED II directive introduced P2P trading of RE, its implementation remains limited in most member states, often restricted to community trading with a single predefined price, as opposed to FEDECOM's vision of a dynamic local market with variable pricing.

***3.1.4 Semantic data model*.** The FEDECOM platform utilizes a Semantic Data Model implemented as an ontology to ensure semantic interoperability between the different modules and components of the platform. This ontology establishes a common vocabulary for information exchange within the FEDECOM platform, allowing for a unified understanding of the various data points and relationships between different energy assets and systems. The Semantic Data Model is essential for enabling the platform to process data from diverse sources, including building energy systems, RE generators, storage systems, and grid infrastructure. It provides a standardized way to represent this information, facilitating communication and data exchange between the various modules responsible for forecasting, optimization, control, and trading. The ontology also enriches the data by providing contextual information and defining relationships between entities, allowing the platform to perform more sophisticated analysis and decision-making. This approach ensures that all components of the FEDECOM platform can effectively communicate and share information, enabling the platform to achieve its goals of optimizing energy use, integrating RES, and facilitating peer-to-peer energy trading within and between energy communities.

***3.1.5 MCDA recommender*.** The MCDA recommender of the FEDECOM platform is an open-source tool designed to help users choose the best energy system configuration based on their specific needs and priorities. It accounts for a variety of factors, such as cost savings, environmental impact, and societal criteria, which are often in conflict with one another. The MCDA recommender uses a weighted summation method, where the user assigns weights to each criterion based on their preferences, which are then used to rank the different system configurations. This allows for a more nuanced and tailored approach to energy system planning, moving beyond simple cost optimization and incorporating factors like sustainability and social impact. The MCDA recommender is intended to be used in conjunction with other FEDECOM tools, such as the EnergyScope planning tool, which performs life-cycle cost (LCC) and environmental impact (LCA) assessments.

### 3.2 Demonstration cases ^1– 4^

The FEDECOM project comprises three large-scale pilots, each with different demonstration (demo) cases. These demo cases are designed to showcase the functionalities and benefits of the FEDECOM platform in diverse scenarios, such as residential communities, industrial facilities, and cross-country energy federations.

***3.2.1 Ur Beroa community and bilbao townhall*.** The Ur Beroa Community and Bilbao Townhall demo case, located in Spain, focuses on integrating RE generation with Power-to-X technologies, specifically hydrogen production. The demo case aims to improve the exploitation of RE by converting excess electricity to hydrogen, which can be stored and utilized later for various applications, such as heating, cooling, and transportation. The system includes a 100 MW photovoltaic (PV) plant, a 20 MW electrolyser for hydrogen production, and a 5 MW/20 MWh hydrogen storage system. This demo case will assess the feasibility and efficiency of integrating hydrogen production and storage into an energy community, promoting self-sufficiency and reducing reliance on the traditional grid. The project aims to achieve cost savings, reduce CO2 emissions, and increase the share of RE in the community's energy mix.

***3.2.2 Puertollano green hydrogen plant*.** The Puertollano Green Hydrogen Plant demo case, also located in Spain, showcases the integration of RE generation with hydrogen production on an industrial scale. The plant features a 2.5 MW electrolyzer and a hydrogen storage system, utilizing electricity generated from a nearby PV plant to produce green hydrogen. The hydrogen produced will be used for various industrial processes, contributing to decarbonization efforts. This demo case will demonstrate the potential of green hydrogen production in reducing the environmental impact of industrial operations, promoting the adoption of RES in industrial sectors, and contributing to a circular economy model for hydrogen utilization.

***3.2.3 TMB barcelona station*.** The TMB Barcelona Station demo case focuses on the integration of green hydrogen into the public transportation sector. The project involves deploying hydrogen-powered buses within the TMB (Transports Metropolitans de Barcelona) fleet, fueled by green hydrogen produced from RES. This demo case aims to showcase the feasibility and benefits of transitioning public transportation systems towards zero-emission solutions. It will assess the operational efficiency of hydrogen buses, the infrastructure requirements for hydrogen refueling, and the potential for reducing the carbon footprint of public transportation systems.

***3.2.4 Swiss residential federation*.** The Swiss Residential Federation demo case focuses on enabling cross-vector demand-side flexibility across multiple communities in Switzerland. This demo case explores community-level conversion opportunities, such as power-to-heat (P2H), and combined heat and power (CHP) coupling, to unlock demand flexibility and offer automated demand response (DR) services to an aggregator for participation in the flexibility market. The pilot leverages existing resources and integrates them with other RES, such as PV, along with thermal storage systems and heat pumps. By coordinating the operation of these assets, the demo case aims to increase local RES penetration and improve power congestion management while offering energy and cost savings for the community.

***3.2.5 Brussels BRICO retail community*.** The Brussels BRICO Retail Community demo case, situated in Belgium, highlights the integration of PV and electric vehicle (EV) charging infrastructure within a retail setting. The project involves installing PV panels on the roof of a BRICO retail store and deploying EV charging points for customers. The demo case aims to demonstrate the potential of retail businesses to become energy prosumers, generating their own RE and offering EV charging services. The project will assess the economic benefits of self-consumption, the impact of EV charging on the local grid, and the potential for offering flexible charging services to support grid stability.

***3.2.6 Voorhout village and besix HQ and eemnes community*.** The Voorhout Village and Besix HQ and Eemnes Community demo cases, located in the Netherlands, demonstrate the cross-country federation of energy communities for enhanced flexibility and grid management. The Voorhout Village demo case involves implementing demand-side management (DSM) measures, such as load shifting for EV charging, to align consumption with local solar generation and reduce peak loads. The Besix HQ and Eemnes Community demo case focuses on aggregating assets within the federation as a virtual power plant (VPP) to participate in ancillary services. By combining resources from different locations, the federated energy communities can unlock additional flexibility, support grid stability, and generate revenue from ancillary services markets. The projects aim to demonstrate the potential of cross-border collaboration in enhancing energy system resilience and maximizing the benefits of RE integration.

### 3.3 Techno-economic assessment ^5^

Techno-Economic assessments in the FEDECOM project are conducted using a combination of modeling tools and methodologies to analyze various aspects of energy systems and their interactions within these communities. These assessments consider design optimization, energy dispatch, LCC, and environmental impact assessment.

The assessments are performed at both the country and community levels, providing a comprehensive understanding of the potential benefits and challenges of implementing the FEDECOM platform. At the country level, the assessments explore the impact of FEDECOM on national energy systems, considering existing energy mix, projected demand, and national policies related to RE and energy communities. At the community level, they focus on optimizing the design and operation of energy systems within specific energy communities, considering local energy resources, demand profiles, and community needs.

The results of the Techno-Economic assessments inform decision-making processes within the FEDECOM project, including selecting suitable and cost-effective technologies for each energy community, developing viable business models, and providing policy recommendations. By providing a comprehensive understanding of the technical, economic, and environmental aspects of the proposed energy community systems, these assessments enable informed decision-making and contribute to the development of sustainable and resilient energy communities.

### 3.4 Cross-border energy trading

Cross-border energy trading in FEDECOM’s BENELUX pilot seeks to demonstrate the feasibility of inter-community energy exchange across national borders. However, evaluating these exchanges is challenging due to the limited maturity of such services. The transposition of RED II article 21, which focuses on peer-to-peer trading, into national legislation in Europe is still incomplete. Therefore, the development of these services needs to be established at the national level before being extended to cross-border exchanges. For example, in the Brussels region, regulations permit peer-to-peer exchanges based on contracts that outline the modalities of the exchange. Potential benefits of cross-border energy trading include increased RE integration, reduced costs, and improved grid resilience.

The FEDECOM platform aims to enable cross-border energy trading through its blockchain-based marketplace and communication protocols. It facilitates secure and transparent energy transactions between energy communities located in different countries, considering factors such as grid constraints, pricing mechanisms, and regulatory requirements. It also leverages standardized protocols for data sharing and energy dispatch, addressing regulatory challenges and data security concerns. Blockchain technology is used here to ensure transparent, verifiable transactions, which are necessary for compliance with national and international standards. Challenges associated with its implementation include regulatory harmonization, data privacy and security, and technical interoperability. Aligning energy regulations and policies across different countries remains a significant challenge. Ensuring data privacy and security in cross-border energy transactions is crucial, particularly when personal data is involved.

## 4. Results

### 4.1 Platform implementation and pilot outcomes

The FEDECOM project has faced challenges in deploying pilot activities due to legal obstacles, public space issues, and supply chain disruptions. To address these issues, the project partners are implementing mitigation strategies, such as direct communication with installation operators, coordinating with stakeholders, and exploring alternative solutions. Despite these challenges, the project has made significant progress in platform implementation and pilot deployment, achieving milestones in developing the platform, defining project requirements, and characterizing pilot sites. The project is committed to mitigating these delays and delivering valuable results for the energy community sector.

### 4.2 Cross-vector energy dispatch modeling outcomes ^6^

The FEDECOM project’s open-source cross-vector energy dispatch model (called Dispa-SET) is being used to simulate and optimize energy flows between different energy vectors, including power, heat, and gas. The model evaluates the performance of energy communities under a range of realistic operational scenarios. The goal is to identify the most efficient and cost-effective strategies for managing energy resources within these communities, taking into account factors such as RE integration, demand flexibility, and grid constraints. One of the expected outcomes of the modeling effort is the identification of optimal locations and utilization rates for grid assets, including batteries and power-to-X conversion plants. The model will also be used to quantify energy distribution losses and to assess the impact of energy trading between communities on grid stability. The Dispa-SET model is coupled with the EnergyScope planning tool, which is used to assess long-term system operation. This coupling enables a comprehensive analysis of both the technical and economic aspects of cross-vector energy dispatch. The project has developed a unique version of the Dispa-SET model, called Dispa-SET-EC, which is specifically tailored for modeling energy communities. The project is also using the Dispa-SET-EC model to simulate and analyze different scenarios in all the pilot sites, focusing on aspects such as optimal dispatch patterns, generation mixes, and curtailments. The results of these simulations will inform the design and operation of the FEDECOM platform and will contribute to the development of best practices for managing energy communities.

### 4.3 Performance and economic viability ^7^

The techno-economic assessment showed that FEDECOM’s platform could yield substantial cost savings while supporting energy autonomy and reduced emissions. Key Performance Indicators (KPIs) are grouped into four main categories: energy efficiency, economic, environmental, and user engagement.

- Energy efficiency KPIs measure improvements in energy consumption patterns, often due to integrating RES. Examples include energy savings, peak load shedding, and predictability of PV production and electricity demand.
- Economic KPIs assess the financial advantages and sustainability of energy community models for local energy markets (LEMs), including energy bill savings, net energy traded, and grid operating margin.
- Environmental KPIs focus on the ecological impact of FEDECOM solutions, with the main KPI being "Avoided CO2 Emissions."
- User engagement KPIs focus on user satisfaction, platform adoption rates, and the effectiveness of user interaction features.

Examples of how these KPIs are calculated and used to evaluate different scenarios and interventions include Bilbao Town Hall's successful transition towards RES and Lugggia, Switzerland's significant increase in self-sufficiency. The FEDECOM project has made significant progress in demonstrating the economic viability of energy communities, showing that investments in new technologies are generally offset by reductions in operating expenses. The project is actively developing business models and exploring replication strategies to ensure long-term sustainability and scalability of its solutions.

## 5. Discussion

### 5.1 Policy implications

The FEDECOM project will enhance EU policymaking in the European Green Deal and Europe for the Digital Age by identifying and addressing regulatory barriers and proposing policy recommendations for broader energy community adoption. The project partners are engaging policymakers via the BRIDGE initiative, gathering best practices to develop policy recommendations, and will disseminate them through publications and briefings. The goal is to contribute to a more sustainable, decentralized, and resilient energy system in Europe. The project aims to address regulatory gaps, particularly in cross-border trading, data privacy, and peer-to-peer transactions, through pilots that promote cross-sector and cross-border energy community interactions.

### 5.2 Recommendations

To improve energy community practices and regulatory frameworks, FEDECOM recommends prioritizing regulatory analysis early in project development, engaging in advocacy activities, supporting the development of local flexibility markets, clarifying regulations related to P2P energy exchange, establishing a regulatory monitoring roadmap, and creating regulatory sandboxes.

Regulatory analysis is crucial in many European countries, as regulations can pose significant obstacles to implementing smart energy solutions. Projects should connect with other EU projects working in the same country and participate in initiatives like BRIDGE to access information and networking opportunities.

Proactive engagement with policymakers and energy regulators is also recommended, as it helps projects stay informed about evolving regulations, anticipate potential barriers, and advocate for necessary adjustments in a timely manner. This includes regular reviews of national and EU-level legislation, engagement with regulatory bodies, and collaboration with other projects to share knowledge and best practices.

Creating regulatory sandboxes allows for experimentation and testing of innovative energy community models and services like P2P trading, fostering innovation and accelerating the development of new energy community solutions. Proactive engagement with policymakers and regulatory bodies, as well as collaboration among project partners and stakeholders, is essential to overcome regulatory barriers and unlock the full potential of energy communities.

## 6. Conclusion

FEDECOM empowers energy communities, allowing residents, businesses, and local authorities to manage their energy use and costs. FEDECOM usage of smart technology like blockchain and artificial intelligence to ensure secure and transparent energy trading between community members entails benefits for citizens that include lower energy bills, cleaner energy, and more control over optimized energy. FEDECOM’s scalable, modular platform and open API design provide a model for replicating the platform’s functionalities in other regions. With the added interoperability of the semantic data model, the FEDECOM framework can adapt to various regulatory environments and technological ecosystems, promoting broad adoption.

The FEDECOM project represents a significant initiative in advancing the development and deployment of energy communities and sector coupling in Europe. The project's comprehensive approach, involving technical characterization of pilot sites, rigorous definition of project requirements, sophisticated platform design, and detailed techno-economic assessment, ensures a robust and impactful solution. FEDECOM's findings and recommendations are expected to contribute to the transition towards a more sustainable and resilient energy system, facilitating the broader adoption of energy community models across Europe.

The FEDECOM project is seeking "follower communities" to join its growing network and replicate its success. Follower communities will have access to a cutting-edge cloud-based platform that offers analytical, modeling, and optimization services to empower energy communities to plan, supervise, and control their integrated local energy systems. They will also receive tailored guidance and support to assess their specific needs and adapt solutions to their local context.

To get involved, visit the project's website at www.fedecom-project.eu, contact the project at fedecom@energies2050.org, or follow the project on social media (LinkedIn and X) for regular updates and insights.

## Disclaimer

The views expressed in this article are those of the author. Publication in Open Research Europe does not imply endorsement by the European Commission.

## Ethics and consent

Ethical approval and consent were not required.

## Data Availability

No data are associated with this article.
