# Supplementary material for: FEDECOM: Enabling cross-border energy exchange by federating energy communities
Source: Open Res Eur. 2025 Feb 10;4:269. Originally published 2024 Dec 19. [Version 2] doi: 10.12688/openreseurope.19146.2 (PMC11814956; doi:10.12688/openreseurope.19146.2)
Supplement: Peer review history

# Reviewer report: Reviewer response for version 2

**DOI:** 10.21956/openreseurope.21248.r51031

**Published:** 2025-02-14

**Authors:** Mian Ibad Ali Shah

**Affiliations:** 1 University of Galway, University Road,, Galway, Ireland

The comments have been addressed.

Where applicable, are recommendations and next steps explained clearly for others to follow? (Please consider whether others in the research community would be able to implement guidelines or recommendations and/or constructively engage in the debate)

Partly

Does the article adequately reference differing views and opinions?

No

Are all factual statements correct, and are statements and arguments made adequately supported by citations?

Partly

Is the rationale for the Open Letter provided in sufficient detail? (Please consider whether existing challenges in the field are outlined clearly and whether the purpose of the letter is explained)

Partly

Is the Open Letter written in accessible language? (Please consider whether all subject-specific terms, concepts and abbreviations are explained)

Yes

Reviewer Expertise:

Reinforcement Learning, Artificial Inteliigence, Peer to Peer Energy Trading, Sustainability, Energy Systems

I confirm that I have read this submission and believe that I have an appropriate level of expertise to confirm that it is of an acceptable scientific standard.

# Reviewer report: Reviewer response for version 2

**DOI:** 10.21956/openreseurope.21248.r51033

**Published:** 2025-02-11

**Authors:** Weiguang Cai

**Affiliations:** 1 Chongqing University, Chongqing, China

The article has been appropriately revised in accordance with my feedback.

Where applicable, are recommendations and next steps explained clearly for others to follow? (Please consider whether others in the research community would be able to implement guidelines or recommendations and/or constructively engage in the debate)

Partly

Does the article adequately reference differing views and opinions?

Yes

Are all factual statements correct, and are statements and arguments made adequately supported by citations?

Partly

Is the rationale for the Open Letter provided in sufficient detail? (Please consider whether existing challenges in the field are outlined clearly and whether the purpose of the letter is explained)

Yes

Is the Open Letter written in accessible language? (Please consider whether all subject-specific terms, concepts and abbreviations are explained)

Yes

Reviewer Expertise:

Building Energy and Carbon Emissions

I confirm that I have read this submission and believe that I have an appropriate level of expertise to confirm that it is of an acceptable scientific standard.

# Reviewer report: Reviewer response for version 1

**DOI:** 10.21956/openreseurope.20717.r49285

**Published:** 2025-01-28

**Authors:** Weiguang Cai

**Affiliations:** 1 Chongqing University, Chongqing, China

This article provides a comprehensive and insightful exposition of the FEDECOM project, showcasing cutting-edge thoughts and practices in cross-border energy exchange. However, there are several areas where the article could benefit from further improvement:

 1. Although the article thoroughly describes technological modules and theoretical frameworks, it lacks substantial data support regarding real-world applications and case analyses. Future research could strengthen the practical effectiveness and feasibility of the theories by providing more data from pilot projects and their outcomes.

 2. The article mentions the policy and regulatory challenges of cross-border energy exchange but does not explore detailed solutions and strategies to overcome these challenges sufficiently. Adding a detailed analysis of the existing policy environment and specific recommendations for improvements would help the project better address these challenges.

 3. The discussion on the potential socio-economic benefits of the project lacks quantitative analysis or concrete evidence to support these conclusions. Future efforts could focus on collecting and presenting specific socio-economic data, such as job growth, economic benefits, and environmental impacts, to enhance the persuasiveness of the arguments.

Where applicable, are recommendations and next steps explained clearly for others to follow? (Please consider whether others in the research community would be able to implement guidelines or recommendations and/or constructively engage in the debate)

Partly

Does the article adequately reference differing views and opinions?

Yes

Are all factual statements correct, and are statements and arguments made adequately supported by citations?

Partly

Is the rationale for the Open Letter provided in sufficient detail? (Please consider whether existing challenges in the field are outlined clearly and whether the purpose of the letter is explained)

Yes

Is the Open Letter written in accessible language? (Please consider whether all subject-specific terms, concepts and abbreviations are explained)

Yes

Reviewer Expertise:

Building Energy and Carbon Emissions

I confirm that I have read this submission and believe that I have an appropriate level of expertise to confirm that it is of an acceptable scientific standard, however I have significant reservations, as outlined above.

# Response

**Published:** 2025-02-06

**Authors:** Zia Lennard

**Affiliations:** R2M Solution, France

I appreciate your constructive feedback.

 I have enhanced the explanation of how FEDECOM's approach to energy management goes beyond existing solutions, providing a more resilient and adaptable system.

 I included detailed empirical results from our pilot projects to demonstrate concrete benefits, such as reductions in line losses and improvements in trading efficiency.

 I have also expanded the socio-economic discussion, adding quantifiable data on job creation and cost savings.

 To address your concern about regulatory engagement, I elaborated on how FEDECOM aligns with and influences evolving EU energy policies.

# Reviewer report: Reviewer response for version 1

**DOI:** 10.21956/openreseurope.20717.r49020

**Published:** 2025-01-22

**Authors:** Osman Zeki Gökçe

**Affiliations:** 1 Istanbul Medipol University, Istanbul, Turkey

Summary: The Open Letter presents the FEDECOM project, an EU-funded project, which aims to enhance the integration and interoperability of energy systems across Europe. It develops a platform using Information and Communication Technologies (ICT) to promote energy efficiency and sectoral integration. Key functionalities range from optimal energy management and peer-to-peer (P2P) trading to promoting demand-side flexibility. Moreover, there are pilot projects showcasing various uses for maintaining grid stability, optimizing renewable energy, and supporting decarbonization efforts.

 Strengths: First of all, the project aligns with the EU Green Deal and Horizon Europe objectives which are to overcome challenges in transitioning to sustainable energy

 systems. Besides this alignment, the project offers innovative modular components each of which seems to be designated with robust methodology and supported by real-world empirical applications.

 Evaluations & Recommendations: 

 - Authors refer to some regulatory and legal challenges of implementing the FEDECOM platform. However, this project is quite extensive and challenges faced especially in application process of functionalities should be elucidated. More important, the discussion of not only foreseen but also unforeseen challenges and how FEDECOM overcomes them would be very illuminating.

 - The letter needs extensive debate of alternative approaches relating to FEDECOM's scope of interest, namely platforms and key functionalities. Without this debate, a reader hardly grasps the very contribution of the FEDECOM project.

 - "Techno-economic assessment" and "performance and economic viability" sections can be enriched by empirical findings of the project to comprehend contributions. In line with the lack of debate of alternative approaches, contribution of the project, compared to what has been done so far, should be highlighted by quantitative data.

Where applicable, are recommendations and next steps explained clearly for others to follow? (Please consider whether others in the research community would be able to implement guidelines or recommendations and/or constructively engage in the debate)

Not applicable

Does the article adequately reference differing views and opinions?

No

Are all factual statements correct, and are statements and arguments made adequately supported by citations?

Partly

Is the rationale for the Open Letter provided in sufficient detail? (Please consider whether existing challenges in the field are outlined clearly and whether the purpose of the letter is explained)

Partly

Is the Open Letter written in accessible language? (Please consider whether all subject-specific terms, concepts and abbreviations are explained)

Yes

Reviewer Expertise:

Interstate Energy Relations; Econometric Modeling of Interstate Energy Relations; Determinants of Inter/Intrastate Conflicts; Twitter and Social Media Analyses.

I confirm that I have read this submission and believe that I have an appropriate level of expertise to confirm that it is of an acceptable scientific standard, however I have significant reservations, as outlined above.

# Response

**Published:** 2025-02-06

**Authors:** Zia Lennard

**Affiliations:** R2M Solution, France

Thank you for your detailed analysis.

 I have integrated more empirical data to support claims regarding grid stability, transaction efficiency, and renewable energy utilization.

 I expanded the technical discussion on FEDECOM’s hybrid consensus mechanism and market-clearing model to emphasize how it differentiates from conventional blockchain-based systems.

 Additionally, I have addressed concerns about scalability and interoperability by illustrating how FEDECOM adapts to different regulatory environments.

 The discussion on policy alignment and standardization efforts has also been extended to highlight our regulatory engagement.

# Reviewer report: Reviewer response for version 1

**DOI:** 10.21956/openreseurope.20717.r49280

**Published:** 2025-01-02

**Authors:** Mian Ibad Ali Shah

**Affiliations:** 1 University of Galway, University Road,, Galway, Ireland

The Open Letter introduces the FEDECOM project, an EU-funded initiative aimed at advancing energy community integration and interoperability across Europe. It highlights a cloud-based platform with modular components for energy management, peer-to-peer (P2P) trading, and demand-side flexibility, alongside pilot projects demonstrating diverse applications for grid stability, renewable energy optimization, and decarbonization.

**Strengths**

1. **Relevance**: The article addresses critical challenges in transitioning to sustainable energy systems, aligning with the EU Green Deal and Horizon Europe objectives.
2. **Technical Contributions**: Innovations like the semantic data model and extended P2P energy trading systems represent significant advances in energy management systems.
3. **Comprehensive Methodology**: The study is supported by detailed techno-economic assessments, ensuring a robust approach to evaluating the viability of energy community models.
4. **Real-World Application**: The demonstration sites provide practical insights into the implementation challenges and benefits of FEDECOM solutions.

**Evaluation and Recommendations**

1. **Insufficient Rationale**
  - **Evaluation**: While the letter identifies general challenges like regulatory barriers and market inefficiencies, it does not provide detailed analysis of specific gaps or how FEDECOM uniquely addresses them.
  - **Recommendation**: Elaborate on key challenges, such as inefficiencies in P2P trading, interoperability limitations, and scalability issues. Clearly articulate how FEDECOM provides innovative solutions.
2. **Limited Engagement with Differing Views**
  - **Evaluation**: The article lacks critical analysis of alternative approaches or debates in the energy community space.
  - **Recommendation**: Discuss contrasting perspectives, such as blockchain limitations for P2P trading or challenges in cross-border exchanges, and compare FEDECOM’s solutions to other EU-funded projects.
3. **Lack of Empirical Support**
  - **Evaluation**: Claims about platform effectiveness, cost savings, and grid stability lack quantitative evidence or sufficient citations.
  - **Recommendation**: Add empirical data from pilot projects or references supporting assertions on line loss reduction, economic benefits, and operational improvements.

**Further Technical Comments:**

1. **P2P Trading Limitations**
  - **Issue**: Heavy reliance on existing blockchain frameworks with little focus on novel contributions.
  - **Recommendation**: Analyze how the platform addresses limitations like transaction costs, scalability, and user adoption. Consider presenting unique auction models or dynamic pricing mechanisms.
2. **Scalability**
  - **Issue**: The discussion on scalability across diverse regulatory and operational environments is insufficient.
  - **Recommendation**: Provide case studies illustrating how the platform adapts to different grid conditions and market demands.
3. **Line Losses**
  - **Issue**: The impact of line losses in energy trading and cross-border exchanges is overlooked.
  - **Recommendation**: Quantify line loss reductions through optimized dispatch or demand response, and include metrics to demonstrate improvements.
4. **Comparative Analysis**
  - **Issue**: The article does not differentiate FEDECOM from similar EU projects.
  - **Recommendation**: Conduct a comparative review to emphasize FEDECOM’s unique advantages, such as better interoperability or cost-effectiveness.
5. **Redundancy with Existing Solutions**
  - **Evaluation**: Some components of the project, such as energy generation forecasting and P2P energy trading, have already been extensively implemented in other projects or platforms. The letter does not explain why these existing solutions were not leveraged.
  - **Recommendation**: Clarify the reasons for not adopting existing solutions. If FEDECOM builds upon or modifies these methods, specify how its approach improves upon or distinguishes itself from established systems.

Where applicable, are recommendations and next steps explained clearly for others to follow? (Please consider whether others in the research community would be able to implement guidelines or recommendations and/or constructively engage in the debate)

Partly

Does the article adequately reference differing views and opinions?

No

Are all factual statements correct, and are statements and arguments made adequately supported by citations?

Partly

Is the rationale for the Open Letter provided in sufficient detail? (Please consider whether existing challenges in the field are outlined clearly and whether the purpose of the letter is explained)

Partly

Is the Open Letter written in accessible language? (Please consider whether all subject-specific terms, concepts and abbreviations are explained)

Yes

Reviewer Expertise:

Reinforcement Learning, Artificial Inteliigence, Peer to Peer Energy Trading, Sustainability, Energy Systems

I confirm that I have read this submission and believe that I have an appropriate level of expertise to confirm that it is of an acceptable scientific standard, however I have significant reservations, as outlined above.

# Response

**Published:** 2025-02-06

**Authors:** Zia Lennard

**Affiliations:** R2M Solution, France

I appreciate your thorough review and insightful feedback. I have addressed your comments by expanding the discussion on inefficiencies in regulatory harmonization and market access, and I have provided empirical data supporting FEDECOM’s impact. I clarified how FEDECOM differs from other initiatives, specifically in its federated approach to cross-border energy trading. Additionally, I elaborated on alternative perspectives, scalability, and the role of P2P trading. The policy section has been expanded to include our active engagement with the BRIDGE initiative, and I have added more data on economic and socio-environmental benefits, including job creation and cost reduction.
